# Supplementary material for: Iterative improvement in the automatic modular design of robot swarms
Source: PeerJ Comput Sci. 2020 Dec 7;6:e322. doi: 10.7717/peerj-cs.322 (PMC7924708; doi:10.7717/peerj-cs.322)
Supplement: Supplemental Information 3 [file peerj-cs-06-322-s003.zip › argos3/doc/api/standalone/a00454_source.html]

ARGoS: plugins/robots/foot-bot/simulator/dynamics2d\_footbot\_model.cpp Source File


- Main Page
- Related Pages
- Namespaces
- Classes
- Files

- File List
- File Members

# plugins/robots/foot-bot/simulator/dynamics2d\_footbot\_model.cpp

Go to the documentation of this file.

```
00001 
00007 #include "dynamics2d_footbot_model.h"
00008 #include "footbot_turret_entity.h"
00009 #include <argos3/plugins/simulator/physics_engines/dynamics2d/dynamics2d_gripping.h>
00010 #include <argos3/plugins/simulator/physics_engines/dynamics2d/dynamics2d_engine.h>
00011 
00012 namespace argos {
00013 
00014    /****************************************/
00015    /****************************************/
00016 
00017    /* P and D constants of the PD controller used for the turret position control. */
00018    static const Real PD_P_CONSTANT = 0.4;
00019    static const Real PD_D_CONSTANT = 0.2;
00020 
00021    static const Real FOOTBOT_RADIUS                   = 0.085036758f;
00022    static const Real FOOTBOT_INTERWHEEL_DISTANCE      = 0.14f;
00023    static const Real FOOTBOT_HEIGHT                   = 0.146899733f;
00024 
00025    static const Real FOOTBOT_MAX_FORCE                = 15.f;
00026    static const Real FOOTBOT_MAX_TORQUE               = 150.f;
00027 
00028    enum FOOTBOT_WHEELS {
00029       FOOTBOT_LEFT_WHEEL = 0,
00030       FOOTBOT_RIGHT_WHEEL = 1
00031    };
00032 
00033    enum ETurretModes {
00034       MODE_OFF,
00035       MODE_PASSIVE,
00036       MODE_SPEED_CONTROL,
00037       MODE_POSITION_CONTROL,
00038    };
00039 
00040    /****************************************/
00041    /****************************************/
00042 
00043    CDynamics2DFootBotModel::CDynamics2DFootBotModel(CDynamics2DEngine& c_engine,
00044                                                     CFootBotEntity& c_entity) :
00045       CDynamics2DMultiBodyObjectModel(c_engine, c_entity),
00046       m_cFootBotEntity(c_entity),
00047       m_cWheeledEntity(m_cFootBotEntity.GetWheeledEntity()),
00048       m_cGripperEntity(c_entity.GetGripperEquippedEntity()),
00049       m_cDiffSteering(c_engine,
00050                       FOOTBOT_MAX_FORCE,
00051                       FOOTBOT_MAX_TORQUE,
00052                       FOOTBOT_INTERWHEEL_DISTANCE),
00053       m_pcGripper(NULL),
00054       m_pcGrippable(NULL),
00055       m_fMass(1.6f),
00056       m_fCurrentWheelVelocity(m_cWheeledEntity.GetWheelVelocities()),
00057       m_unLastTurretMode(m_cFootBotEntity.GetTurretEntity().GetMode()) {
00058       RegisterAnchorMethod<CDynamics2DFootBotModel>(
00059          GetEmbodiedEntity().GetOriginAnchor(),
00060          &CDynamics2DFootBotModel::UpdateOriginAnchor);
00061       RegisterAnchorMethod<CDynamics2DFootBotModel>(
00062          GetEmbodiedEntity().GetAnchor("turret"),
00063          &CDynamics2DFootBotModel::UpdateTurretAnchor);
00064       RegisterAnchorMethod<CDynamics2DFootBotModel>(
00065          GetEmbodiedEntity().GetAnchor("perspective_camera"),
00066          &CDynamics2DFootBotModel::UpdatePerspectiveCameraAnchor);
00067       /* Create the actual body with initial position and orientation */
00068       m_ptActualBaseBody =
00069          cpSpaceAddBody(GetDynamics2DEngine().GetPhysicsSpace(),
00070                         cpBodyNew(m_fMass,
00071                                   cpMomentForCircle(m_fMass,
00072                                                     0.0f,
00073                                                     FOOTBOT_RADIUS + FOOTBOT_RADIUS,
00074                                                     cpvzero)));
00075       const CVector3& cPosition = GetEmbodiedEntity().GetOriginAnchor().Position;
00076       m_ptActualBaseBody->p = cpv(cPosition.GetX(), cPosition.GetY());
00077       CRadians cXAngle, cYAngle, cZAngle;
00078       GetEmbodiedEntity().GetOriginAnchor().Orientation.ToEulerAngles(cZAngle, cYAngle, cXAngle);
00079       cpBodySetAngle(m_ptActualBaseBody, cZAngle.GetValue());
00080       /* Create the actual body shape */
00081       m_ptBaseShape =
00082          cpSpaceAddShape(GetDynamics2DEngine().GetPhysicsSpace(),
00083                          cpCircleShapeNew(m_ptActualBaseBody,
00084                                           FOOTBOT_RADIUS,
00085                                           cpvzero));
00086       m_ptBaseShape->e = 0.0; // No elasticity
00087       m_ptBaseShape->u = 0.7; // Lots of friction
00088       /* This shape is grippable */
00089       m_pcGrippable = new CDynamics2DGrippable(GetEmbodiedEntity(),
00090                                                m_ptBaseShape);
00091       /* Constrain the actual base body to follow the diff steering control */
00092       m_cDiffSteering.AttachTo(m_ptActualBaseBody);
00093       /* Add the body so that the default methods work as expected */
00094       AddBody(m_ptActualBaseBody, cpvzero, 0, FOOTBOT_HEIGHT);
00095       /* Create the gripper body */      
00096       m_ptActualGripperBody =
00097          cpSpaceAddBody(GetDynamics2DEngine().GetPhysicsSpace(),
00098                         cpBodyNew(m_fMass / 20.0,
00099                                   cpMomentForCircle(m_fMass,
00100                                                     0.0f,
00101                                                     FOOTBOT_RADIUS + FOOTBOT_RADIUS,
00102                                                     cpvzero)));
00103       m_ptActualGripperBody->p = cpv(cPosition.GetX(), cPosition.GetY());
00104       cpBodySetAngle(m_ptActualGripperBody,
00105                      cZAngle.GetValue() +
00106                      m_cFootBotEntity.GetTurretEntity().GetRotation().GetValue());
00107       /* Create the gripper shape */
00108       cpShape* ptGripperShape = 
00109          cpSpaceAddShape(GetDynamics2DEngine().GetPhysicsSpace(),
00110                          cpCircleShapeNew(m_ptActualGripperBody,
00111                                           0.01f,
00112                                           cpv(FOOTBOT_RADIUS, 0.0f)));
00113       m_pcGripper = new CDynamics2DGripper(GetDynamics2DEngine(),
00114                                            m_cGripperEntity,
00115                                            ptGripperShape);
00116       /* Constrain the actual gripper body to follow the actual base body */
00117       m_ptBaseGripperLinearMotion =
00118          cpSpaceAddConstraint(GetDynamics2DEngine().GetPhysicsSpace(),
00119                               cpPivotJointNew2(m_ptActualBaseBody,
00120                                                m_ptActualGripperBody,
00121                                                cpvzero,
00122                                                cpvzero));
00123       m_ptBaseGripperAngularMotion = cpSpaceAddConstraint(GetDynamics2DEngine().GetPhysicsSpace(),
00124                                                           cpGearJointNew(m_ptActualBaseBody,
00125                                                                          m_ptActualGripperBody,
00126                                                                          0.0f,
00127                                                                          1.0f));
00128       m_ptBaseGripperAngularMotion->maxBias = 0.0f; /* disable joint correction */
00129       m_ptBaseGripperAngularMotion->maxForce = FOOTBOT_MAX_TORQUE; /* limit the dragging torque */
00130       /* Add the gripper body so that the default methods work as expected */
00131       AddBody(m_ptActualGripperBody, cpvzero, 0, FOOTBOT_HEIGHT);
00132       /* Switch to active mode if necessary */
00133       if(m_unLastTurretMode == MODE_SPEED_CONTROL ||
00134          m_unLastTurretMode == MODE_POSITION_CONTROL) {
00135          TurretActiveToPassive();
00136       }
00137    }
00138 
00139    /****************************************/
00140    /****************************************/
00141 
00142    CDynamics2DFootBotModel::~CDynamics2DFootBotModel() {
00143       delete m_pcGripper;
00144       delete m_pcGrippable;
00145       switch(m_unLastTurretMode) {
00146          case MODE_OFF:
00147          case MODE_PASSIVE:
00148             cpSpaceRemoveConstraint(GetDynamics2DEngine().GetPhysicsSpace(), m_ptBaseGripperLinearMotion);
00149             cpSpaceRemoveConstraint(GetDynamics2DEngine().GetPhysicsSpace(), m_ptBaseGripperAngularMotion);
00150             cpConstraintFree(m_ptBaseGripperLinearMotion);
00151             cpConstraintFree(m_ptBaseGripperAngularMotion);
00152             break;
00153          case MODE_POSITION_CONTROL:
00154          case MODE_SPEED_CONTROL:
00155             cpSpaceRemoveConstraint(GetDynamics2DEngine().GetPhysicsSpace(), m_ptBaseGripperLinearMotion);
00156             cpSpaceRemoveConstraint(GetDynamics2DEngine().GetPhysicsSpace(), m_ptGripperControlAngularMotion);
00157             cpConstraintFree(m_ptBaseGripperLinearMotion);
00158             cpConstraintFree(m_ptGripperControlAngularMotion);
00159             cpBodyFree(m_ptControlGripperBody);
00160             break;
00161       }
00162       m_cDiffSteering.Detach();
00163    }
00164 
00165    /****************************************/
00166    /****************************************/
00167 
00168    void CDynamics2DFootBotModel::MoveTo(const CVector3& c_position,
00169                                         const CQuaternion& c_orientation) {
00170       /* Release grippers and grippees */
00171       m_pcGripper->Release();
00172       m_pcGrippable->ReleaseAll();
00173       /* Move robot */
00174       CDynamics2DMultiBodyObjectModel::MoveTo(c_position,
00175                                               c_orientation);
00176    }
00177 
00178    /****************************************/
00179    /****************************************/
00180 
00181    void CDynamics2DFootBotModel::Reset() {
00182       /* Zero speed and applied forces of base control body */
00183       m_cDiffSteering.Reset();
00184       /* Release grippers and gripees */
00185       m_pcGripper->Release();
00186       m_pcGrippable->ReleaseAll();
00187       /* Switch to turret passive mode if needed */
00188       if(m_unLastTurretMode == MODE_SPEED_CONTROL ||
00189          m_unLastTurretMode == MODE_POSITION_CONTROL) {
00190          TurretActiveToPassive();
00191          m_unLastTurretMode = MODE_OFF;
00192          GetEmbodiedEntity().DisableAnchor("turret");
00193       }
00194       /* Reset the rest */
00195       CDynamics2DMultiBodyObjectModel::Reset();
00196    }
00197 
00198    /****************************************/
00199    /****************************************/
00200 
00201    void CDynamics2DFootBotModel::CalculateBoundingBox() {
00202       GetBoundingBox().MinCorner.SetX(m_ptBaseShape->bb.l);
00203       GetBoundingBox().MinCorner.SetY(m_ptBaseShape->bb.b);
00204       GetBoundingBox().MinCorner.SetZ(GetDynamics2DEngine().GetElevation());
00205       GetBoundingBox().MaxCorner.SetX(m_ptBaseShape->bb.r);
00206       GetBoundingBox().MaxCorner.SetY(m_ptBaseShape->bb.t);
00207       GetBoundingBox().MaxCorner.SetZ(GetDynamics2DEngine().GetElevation() + FOOTBOT_HEIGHT);
00208    }
00209 
00210    /****************************************/
00211    /****************************************/
00212 
00213    void CDynamics2DFootBotModel::UpdateFromEntityStatus() {
00214       /* Do we want to move? */
00215       if((m_fCurrentWheelVelocity[FOOTBOT_LEFT_WHEEL] != 0.0f) ||
00216          (m_fCurrentWheelVelocity[FOOTBOT_RIGHT_WHEEL] != 0.0f)) {
00217          m_cDiffSteering.SetWheelVelocity(m_fCurrentWheelVelocity[FOOTBOT_LEFT_WHEEL],
00218                                           m_fCurrentWheelVelocity[FOOTBOT_RIGHT_WHEEL]);
00219       }
00220       else {
00221          /* No, we don't want to move - zero all speeds */
00222          m_cDiffSteering.Reset();
00223       }
00224       /* Update turret structures if the state changed state in the last step */
00225       if(m_cFootBotEntity.GetTurretEntity().GetMode() != m_unLastTurretMode) {
00226          /* Enable or disable the anchor */
00227          if(m_cFootBotEntity.GetTurretEntity().GetMode() != MODE_OFF) {
00228             GetEmbodiedEntity().EnableAnchor("turret");
00229          }
00230          else {
00231             GetEmbodiedEntity().DisableAnchor("turret");
00232          }
00233          /* Manage the thing like a state machine */
00234          switch(m_unLastTurretMode) {
00235             case MODE_OFF:
00236             case MODE_PASSIVE:
00237                switch(m_cFootBotEntity.GetTurretEntity().GetMode()) {
00238                   case MODE_POSITION_CONTROL:
00239                   case MODE_SPEED_CONTROL:
00240                      TurretPassiveToActive();
00241                      break;
00242                   case MODE_OFF:
00243                   case MODE_PASSIVE:
00244                      break;
00245                }
00246                break;
00247             case MODE_SPEED_CONTROL:
00248             case MODE_POSITION_CONTROL:
00249                switch(m_cFootBotEntity.GetTurretEntity().GetMode()) {
00250                   case MODE_OFF:
00251                   case MODE_PASSIVE:
00252                      TurretActiveToPassive();
00253                      break;
00254                   case MODE_POSITION_CONTROL:
00255                   case MODE_SPEED_CONTROL:
00256                      break;
00257                }
00258                break;
00259          }
00260          /* Save the current mode for the next time step */
00261          m_unLastTurretMode = m_cFootBotEntity.GetTurretEntity().GetMode();
00262       }
00263       /* Update the turret data */
00264       switch(m_unLastTurretMode) {
00265          /* Position control mode is implemented using a PD controller */
00266          case MODE_POSITION_CONTROL: {
00267             Real fCurRotErr = NormalizedDifference(
00268                m_cFootBotEntity.GetTurretEntity().GetDesiredRotation(),
00269                NormalizedDifference(
00270                   CRadians(m_ptActualGripperBody->a),
00271                   CRadians(m_ptActualBaseBody->a))).GetValue();
00272             m_ptControlGripperBody->w =
00273                m_cDiffSteering.GetAngularVelocity() +
00274                (PD_P_CONSTANT * fCurRotErr +
00275                 PD_D_CONSTANT * (fCurRotErr - m_fPreviousTurretAngleError) * GetDynamics2DEngine().GetInverseSimulationClockTick());
00276             m_fPreviousTurretAngleError = fCurRotErr;
00277             break;
00278          }
00279          case MODE_SPEED_CONTROL:
00280             m_ptControlGripperBody->w =
00281                m_cDiffSteering.GetAngularVelocity() +
00282                m_cFootBotEntity.GetTurretEntity().GetDesiredRotationSpeed();
00283             break;
00284          case MODE_OFF:
00285          case MODE_PASSIVE:
00286             if(m_cGripperEntity.IsGripping() &&
00287                m_cGripperEntity.IsLocked()) {
00288                m_ptBaseGripperAngularMotion->maxForce = 0.0001f; /* limit the dragging torque */
00289             }
00290             else {
00291                m_ptBaseGripperAngularMotion->maxForce = FOOTBOT_MAX_TORQUE; /* limit the dragging torque */
00292             }
00293             break;
00294       }
00295    }
00296 
00297    /****************************************/
00298    /****************************************/
00299 
00300    void CDynamics2DFootBotModel::TurretPassiveToActive() {
00301       /* Delete constraints to actual base body */
00302       cpSpaceRemoveConstraint(GetDynamics2DEngine().GetPhysicsSpace(), m_ptBaseGripperAngularMotion);
00303       cpConstraintFree(m_ptBaseGripperAngularMotion);
00304       /* Create gripper control body */
00305       m_ptControlGripperBody = cpBodyNew(INFINITY, INFINITY);
00306       /* Create angular constraint from gripper control body to gripper actual body */
00307       m_ptGripperControlAngularMotion = cpSpaceAddConstraint(GetDynamics2DEngine().GetPhysicsSpace(),
00308                                                              cpGearJointNew(m_ptActualGripperBody,
00309                                                                             m_ptControlGripperBody,
00310                                                                             0.0f,
00311                                                                             1.0f));
00312       m_ptGripperControlAngularMotion->maxBias = 0.0f; /* disable joint correction */
00313       m_ptGripperControlAngularMotion->maxForce = FOOTBOT_MAX_TORQUE; /* limit the dragging torque */
00314    }
00315 
00316    /****************************************/
00317    /****************************************/
00318 
00319    void CDynamics2DFootBotModel::TurretActiveToPassive() {
00320       /* Delete constraint from actual gripper body to gripper control body */
00321       cpSpaceRemoveConstraint(GetDynamics2DEngine().GetPhysicsSpace(), m_ptGripperControlAngularMotion);
00322       cpConstraintFree(m_ptGripperControlAngularMotion);
00323       /* Delete control body */
00324       cpBodyFree(m_ptControlGripperBody);
00325       /* Create constraints from actual gripper body to actual base body */
00326       m_ptBaseGripperAngularMotion = cpSpaceAddConstraint(GetDynamics2DEngine().GetPhysicsSpace(),
00327                                                           cpGearJointNew(m_ptActualBaseBody,
00328                                                                          m_ptActualGripperBody,
00329                                                                          0.0f,
00330                                                                          1.0f));
00331       m_ptBaseGripperAngularMotion->maxBias = 0.0f; /* disable joint correction */
00332       m_ptBaseGripperAngularMotion->maxForce = FOOTBOT_MAX_TORQUE; /* limit the dragging torque */
00333    }
00334 
00335    /****************************************/
00336    /****************************************/
00337 
00338    void CDynamics2DFootBotModel::UpdateOriginAnchor(SAnchor& s_anchor) {
00339       s_anchor.Position.SetX(m_ptActualBaseBody->p.x);
00340       s_anchor.Position.SetY(m_ptActualBaseBody->p.y);
00341       s_anchor.Orientation.FromAngleAxis(CRadians(m_ptActualBaseBody->a), CVector3::Z);
00342    }
00343 
00344    /****************************************/
00345    /****************************************/
00346 
00347    void CDynamics2DFootBotModel::UpdateTurretAnchor(SAnchor& s_anchor) {
00348       s_anchor.Position.SetX(m_ptActualGripperBody->p.x);
00349       s_anchor.Position.SetY(m_ptActualGripperBody->p.y);
00350       s_anchor.Orientation.FromAngleAxis(CRadians(m_ptActualGripperBody->a), CVector3::Z);
00351       s_anchor.OffsetOrientation.FromAngleAxis(
00352          NormalizedDifference(
00353             CRadians(m_ptActualGripperBody->a),
00354             CRadians(m_ptActualBaseBody->a)),
00355          CVector3::Z);
00356    }
00357 
00358    /****************************************/
00359    /****************************************/
00360 
00361    void CDynamics2DFootBotModel::UpdatePerspectiveCameraAnchor(SAnchor& s_anchor) {
00362       s_anchor.Position.SetX(m_ptActualBaseBody->p.x + s_anchor.OffsetPosition.GetX());
00363       s_anchor.Position.SetY(m_ptActualBaseBody->p.y + s_anchor.OffsetPosition.GetY());
00364       s_anchor.Orientation =
00365          s_anchor.OffsetOrientation *
00366          CQuaternion(CRadians(m_ptActualBaseBody->a), CVector3::Z);
00367    }
00368 
00369    /****************************************/
00370    /****************************************/
00371 
00372    REGISTER_STANDARD_DYNAMICS2D_OPERATIONS_ON_ENTITY(CFootBotEntity, CDynamics2DFootBotModel);
00373 
00374    /****************************************/
00375    /****************************************/
00376 
00377 }
```

---

Generated on 10 Jul 2018 for ARGoS by 
 1.6.1
